# Preliminary Development of a Screening Tool for Pre-Clinical Dysphagia in Community Dwelling Older Adults

**DOI:** 10.3390/geriatrics3040090

**Published:** 2018-12-07

**Authors:** Aarthi Madhavan, Giselle D. Carnaby, Karishma Chhabria, Michael A. Crary

**Affiliations:** 1 Department of Communication Sciences and Disorders, The Pennsylvania State University, University Park, PA 16802, USA; 2 Swallowing Research Laboratory, School of Communication Sciences and Disorders, University of Central Florida, Orlando, FL 32816, USA; giselle.carnaby@ucf.edu (G.D.C.); Michael.crary@ucf.edu (M.A.C.); 3 Department of Medicine, Baylor College of Medicine, Houston, TX 77030, USA; karishma.chhabria@bcm.edu

**Keywords:** pre-clinical dysphagia, screening, patient reported outcomes, tool development

## Abstract

Evidence suggests that community dwelling older adults (CDOA) are at risk for dysphagia (swallowing difficulties). Dysphagia is often unidentified until related morbidities like under nutrition or pneumonia occur. These cases of unidentified dysphagia, prior to any clinical intervention, may be termed ‘pre-clinical dysphagia’. Identifying pre-clinical dysphagia is challenged by the lack of validated tools appropriate for CDOA. This study addresses preliminary development of a novel patient reported outcome (PRO) screening tool for pre-clinical dysphagia. Initially, 34 questions were developed from literature review and expert opinion. Following pilot testing (n = 53), the questionnaire was revised and tested on 335 additional CDOA. Face validity, content validity, item analysis, reliability (internal consistency), and construct validity (exploratory factor analysis) measures were completed. Psychometric validation resulted in a 17-question PRO tool. Construct analysis identified a three-factor model that explained 67.345% of the variance. Emergent factors represented swallowing effort, physical function, and cognitive function. The results revealed strong construct validity and internal consistency (Cronbach’s α = 0.90). A novel, simple PRO incorporating multiple function domains associated with aging demonstrated strong preliminary psychometric properties. This tool is more comprehensive and aging-focused than existing dysphagia screening tools. Inclusion of multiple domains may be key in early identification of pre-clinical dysphagia.

## 1. Introduction 

In the aging population, dysphagia (swallowing disorder) and its associated morbidities lead to increasing healthcare complications, including malnutrition, dehydration, aspiration pneumonia, falls, increased hospitalizations, and mortality [1,2]. In fact, the Centers for Disease Control (CDC) ranks mortality from food and liquid aspiration as a top-15 cause of death in US adults older than 65 years and this ranking is higher with advancing age [3]. Negative health consequences from dysphagia are well studied in individuals with clinical diseases like stroke or head and neck cancer [4,5]; however, less is known about community dwelling older adults (CDOA), who are not otherwise in need of medical or skilled care. The number of elderly individuals in the US is expected to reach 72.1 million by 2030. Of these, 11.8 million older adults live alone and may be considered CDOA [3,6]. A growing body of evidence suggests CDOA are at risk for developing dysphagia with potential prevalence rates as high as 35% [7,8,9,10,11,12,13,14,15,16,17,18,19,20,21]. These conservative estimates suggest that within the US alone, CDOA at risk for developing dysphagia may be in excess of 4 million. 

Community dwelling older adults do not routinely seek help for swallowing limitations [11,14,15,18]. Instead, these individuals consider difficulty swallowing as a natural part of aging and employ compensatory strategies such as modifying their diets or avoiding/reducing their dietary intake to manage swallowing difficulties [16,18,19]. However, such strategies may lead to declines in nutrition and general health, along with an associated increase in frailty [16,18,19]. Furthermore, reported dysphagia in CDOA can remain undetected until individuals are hospitalized or diagnosed with dysphagia-related morbidities such as pneumonia [19]. For example, in a recent three-year longitudinal study on dysphagia in the CDOA, participants who died from pneumonia perceived worse swallowing ability at baseline measurement, prior to the onset of any dysphagia-related morbidities [22]. These cases of unidentified dysphagia (prior to an individual seeking clinical assistance) may be appropriately termed ‘pre-clinical dysphagia’. 

A systematic review by Madhavan et al. [23] found a mean prevalence of 15% dysphagia among CDOA. However, estimates of prevalence are highly variable and studies have not employed assessment methods specific to CDOA. Identified risk factors for dysphagia in CDOA have included; a history of diagnosed clinical disease, age > 70 years, cognitive decline, and physical frailty, including a reduced ability to carry out independent activities of daily living. Several factors have been associated with physical and cognitive decline, including nutrition and social factors like living conditions and family support [24,25,26]. Additionally, tooth loss and xerostomia or oral dryness have been shown to be associated with difficulty chewing hard materials and transporting these foods through the oral cavity [16,17,27]. Despite these identified associations, a major barrier to estimating the scope of dysphagia in the CDOA is the absence of an assessment tool specifically validated for this population. Given the potential scope of dysphagia and related morbidities in CDOA, it is critical that valid and reliable population-specific measure be developed. 

Only three dysphagia assessment tools have been developed and validated for use in healthy older adults: The Volume Viscosity Swallow Test (V-VST) [28], The Sydney Swallowing Questionnaire (SSQ) [29], and the Eating Assessment Tool-10 (EAT-10) [30]. While validated using older populations, all three tools present limitations for use with the pre-clinical dysphagia population. Specifically, all three are designed to evaluate the severity of dysphagia in symptomatic individuals, and the tools do not address a wide range of factors reportedly contributing to swallowing limitations in this population (e.g., oral health, cognitive decline, physical issues). Early recognition of symptoms known to be associated with swallowing difficulties may play an essential role in screening for pre-clinical dysphagia. This paper describes the initial development of a patient reported outcome (PRO) screening tool for the early identification of dysphagia risk in the CDOA. Preliminary psychometric analyses are presented for this novel yet simple PRO dysphagia screening tool for CDOA populations. Being a PRO, it is envisioned that this tool could be part of annual medical check-ups in doctors’ offices, easily handed out at clinics or even adult day care centers, etc. 

## 2. Design and Methods

To achieve a comprehensive screener, creation proceeded through two phases. These were: (1) initial questionnaire development and testing using a pilot sample of CDOA; and (2) psychometric analysis including construct validity and exploratory factor analysis (EFA) on a larger sample of CDOA. The designated University Institutional Review Board approved the study.

### 2.1. Phase 1–Tool Development and Pilot Application 

#### 2.1.1. Participant Sample 

Participants were recruited from a web-based research site (www.researchmatch.org) that connects interested volunteers with appropriate research studies. Participants were recruited from across the USA. Once a subject agreed to participate via online informed consent, they received an anonymous survey link, via the survey-hosting site, Qualtrics (www.Qualtrics.com). The criterion for inclusion was: >60 years of age with no gender preference. This age cut-off was selected because evidence from swallowing, nutrition, oral health, and physical abilities suggest that age-related changes occur in the sixth decade or even earlier [4,5,31,32,33]. Exclusion criteria included: any reported medical condition known to cause dysphagia, e.g., history of stroke, Parkinson’s disease, dementia, etc. [4,5]

#### 2.1.2. Tool Development

**Figure 1:**
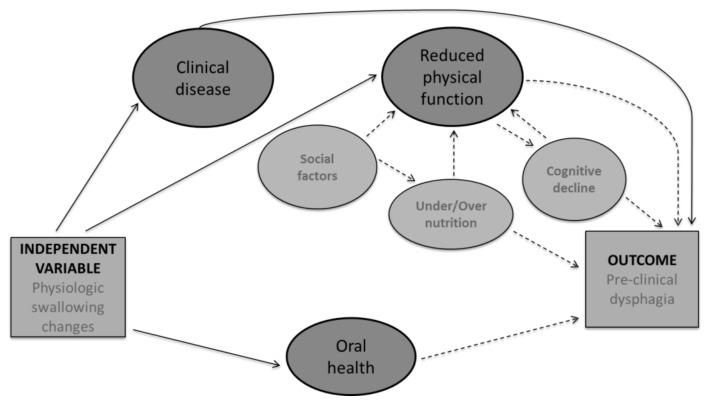
Proposed conceptual framework for the development of pre-clinical dysphagia in the CDOA. Proposed theoretical model for the development of pre-clinical dysphagia in CDOA. Solid lines represent known causes while dashed lines represent reported associations.

In older adults, swallowing undergoes several physiologic changes, based on reduced muscle strength, reduced range of motion, and decline in reserve capacity [1,5,8]. Despite these changes, not all older adults develop dysphagia. To better understand these relationships, a conceptual framework was employed to guide tool content development based on available research of risk factors for dysphagia in CDOA (Figure 1) [23]. In the proposed conceptual framework, a history of clinical disease, a known cause of dysphagia with increased prevalence in older adults was considered an essential part on the development of pre-clinical dysphagia [4,34,35]. Reduced physical functioning including difficulty with activities of daily living and pre-frailty leading to frailty is frequently associated with a decline in physical functioning and included in the theoretical framework. Several factors contribute to reduced physical functioning in the elderly; including cognitive decline [36,37], under/over nutrition [33,38], and social factors such as available support [39,40]. All of these factors were considered for item development. Lastly, oral health including dentition and xerostomia (oral dryness) has been associated with dysphagia and was included in the conceptual framework and item development. However, when considering these factors as contributing to pre-clinical dysphagia, the majority of the evidence is in the form of associative data. Therefore, in the conceptual framework, known causes for dysphagia are represented by a solid line and associations are presented via dashed lines. Items based on these factors were developed by the authors. The item pool was reviewed by two speech language pathologists and two gerontologists not associated with the study. This review determined item appropriateness, and gauged face validity. Raters graded each item for theoretical relevance, clinical significance, wording, and cohesiveness. Following this review, the pilot survey contained 34 items representing five broad categories drawn from the conceptual framework (see item examples in Table 1). Pilot field testing was completed using the first 54 recruited participants. Item analysis techniques were applied to the results of the field test to determine the contribution of each item to the overall questionnaire. Item selection was made based on item to total correlation (>0.04) and item Cronbach’s Alpha (>0.85) denoting adequacy for inclusion.

#### 2.1.3. Statistical Analysis

Statistical analysis of the tool was conducted descriptively and analytically at each phase of development. Statistical analysis only included the data for subjects who completed the questionnaire. Descriptive analyses included a review of unengaged responses, normality testing, analysis of response categories, classical item difficulty, and inter-item correlations. Unengaged participants were determined based on a lack of variance in responses (Mean and SD = 0). Inter-item correlations were used to evaluate item redundancy (r > 0.9) and poor fit (r < 0.3). Concordance of expert ratings across each of the evaluation criteria was evaluated using intraclass correlations (ICC). Questionnaire internal consistency was evaluated using Cronbach’s alpha. Item difficulty and item discrimination was used to evaluate participants’ perception of the items. Classical item difficulty was determined by the mean response value for polytomous items or proportion of ‘yes’ responses for dichotomous items, reported as a scale of 0–1. Item difficulty was then interpreted as the probability of responding in the keyed direction for each item. The standard item difficulty cut off (range: 0 to 1) was used to categorize items as very easy (>0.91), easy (0.76–0.90), optimally difficult (0.26–0.75), difficult (0.11–0.25), or very difficult (<0.10) [41]. Item discrimination was employed to identify the relationship between each item and to determine which questions differentiated dysphagic vs. non-dysphagic respondents. An item was considered to discriminate when the perceived difficulty index differed significantly between groups according to effect size calculation [41,42]. To examine construct validity, exploratory factor analysis (EFA) was completed with principal component analysis (PCA) with oblique (direct oblimin) rotation. All statistical analyses were completed using Microsoft Excel and IBM’s SPSS Statistical Package 22.0 (IBM Corporation, Armonk, NY, USA, 2013). 

### 2.2. Results: Pilot Application

One participant was removed for unengaged responses to the items, resulting in a sample of N = 53. Participants were primarily in the 60–70 year age range (90%), with 75% of the sample being women. Overall the face validity of the items were considered adequate for inclusion (average ICC (2, k) 0.44). Evaluation of normality identified that in 22 items, the response category ‘all the time’ was not selected. Subsequently, items were collapsed and analyzed using a four ordered-category response scale (never, rarely, sometimes, and often). Evaluation of inter-item correlations revealed three items, demonstrating item redundancy and two other items with statistically weak inter-item correlations. Following item removal, the revised survey contained 28 questions across the same 5 model driven categories—9 swallowing questions, 8 oral health questions, 5 physical abilities questions, 3 cognitive abilities questions, and 3 social support related questions. 

### 2.3. Phase 2—Psychometric Evaluation of Revised Questionnaire 

#### Participant Sample and Statistical Analysis

The revised 28-item questionnaire was evaluated using data from a new sample of 335 CDOA, recruited and enrolled using the methodology described for Phase 1. Analysis of the larger sample included descriptive statistics, item analysis, reliability analysis, and construct validity through exploratory factor analysis (EFA). Differences in participant responses were examined by dividing the participants into those who reported modifying their diet due to difficulty swallowing (dysphagic), and those who did not (non-dysphagic). To evaluate item performance between these two subgroups, items with difficulty values that were reflective of more positive vs. negative experiences with swallowing were compared. Items demonstrating a difference of >2.5 standard deviations between dysphagic and non-dysphagic CDOA responses were considered a meaningful difference between the groups. To further evaluate the strength of the difference between dysphagic and non-dysphagic, effect sizes were calculated (Hedge’s g).

### 2.4. Results: Phase 2 

#### 2.4.1. Participant Sample

In total, 770 potential participants were contacted regarding this study. Of these, 570 (74%) agreed to participate in the study and were sent the anonymous survey link. Reasons for refusal included: not interested in the study (63%), confusion regarding study eligibility (23%), not interested in participating without compensation (7%), or another reason (7%). Completion rate for the survey was 63% (358). Once started, only three participants abandoned the questionnaire prior to completion. As the questionnaire design required a response from each question before proceeding to the next, no missing data resulted. Of the 358 responses received, 20 participants did not meet inclusion criteria and 3 participants’ patterns of response were identified as unengaged. These 23 participants were removed prior to analysis. 

#### 2.4.2. Descriptive Results

Data from 335 participants was used in phase 2 analysis. Eighty percent (80%) of the sample were aged between 60–70 years, and 70% of the sample comprised women. The entire sample had a high school degree or higher; with 44% holding postgraduate degrees. Eighty percent of the sample reported current or a history of one or more medical conditions including but not limited to hypertension, GERD, arthritis, and diabetes. Sixty-eight percent (68%) of participants lived with family and the remainder lived alone. Forty percent (40%) of the sample reported some difficulty with one or more aspects of swallowing. Most reported difficulties included increased effort with swallowing solids (36.7%) and a sensation of food getting stuck in the throat (42%), 11% reported modifying their diet or avoiding food items due to difficulty swallowing (Table 2). 

#### 2.4.3. Item Analysis

**Figure 2:**
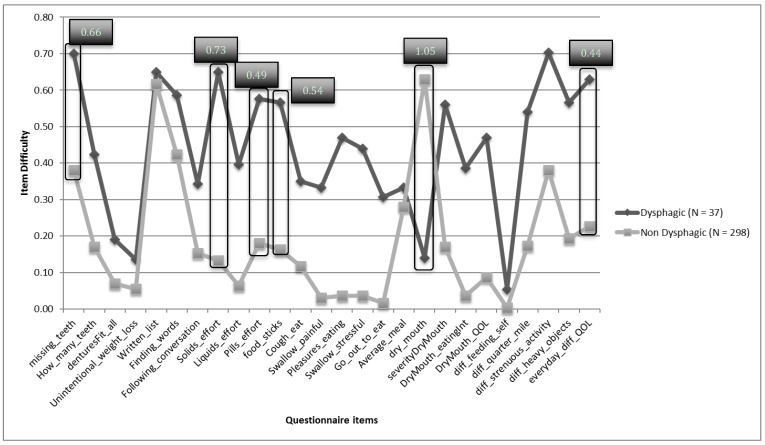
Item discrimination graph depicting the difference in perception of item difficulty between participants with and without dysphagia. Items that provided the most meaningful difference are circled, with effect size (Hedge’s g) identified.

Classic item difficulty revealed a third of the items were perceived as difficult (35.71%) and another third as very difficult (39.29%), 21.43% of items were perceived as optimally difficult, and 3.6% of items were perceived as easy. Item discrimination was used to determine which items best differentiated between participants with and without reported swallowing difficulty (Figure 2). Item difficulty levels were compared between the groups and those items demonstrating a significant effect difference (Hedge’s g) were considered most meaningful. Six items provided the most meaningful difference with effect sizes ranging from 0.44 to 1.05. These items queried missing teeth, effort of swallowing solids, effort of swallowing pills, experience of food sticking, experience of a dry mouth, and how everyday difficulties impacted their quality of life. All but one question (experience of dry mouth) was perceived as more difficult among persons without swallowing problems compared to people with swallowing problems.

#### 2.4.4. Inter-Item Correlations

Inter-item correlations were employed to evaluate item redundancy. Items demonstrated strong correlations (>0.9) were reviewed and considered for removal from the questionnaire. Two items relating to dry mouth (dry mouth severity and dry mouth interference with QOL) were strongly correlated (r = 0.915), indicating redundancy. As a result, only the question regarding dry mouth severity was retained as it was significant in differentiating between participants with or without swallowing difficulty. Similarly, two other questions regarding dentition (are you missing teeth and how many teeth) were collapsed to reduce redundancy, resulting in 22 items analyzed for reliability and construct validity. 

#### 2.4.5. Reliability–Internal Consistency

Internal consistency via Cronbach’s alpha was evaluated as a measure of determining if the items were reliable, i.e., measured the same aspect. Poor inter-item correlations (<0.3) that worsened the questionnaire’s internal consistency were considered for removal. Reliability analysis resulted in the removal of five items (dentition, unintentional weight loss, need to use memory aids, difficulty self-feeding, and time to eat an average meal). This resulted in a 17-item tool with increase in internal consistency from α = 0.89 to α = 0.90. 

#### 2.4.6. Exploratory Factor Analysis (EFA)

To identify the underlying structure of the CDOA screener, responses on the 17-item questionnaire were submitted to EFA. Oblique rotation was applied due to anticipated inter-correlation between swallow-related components. To identify the final factor solution both scree plot and significantly loaded items were evaluated. The Kaiser–Meyer–Olkin (KMO) score = 0.917 verified the sampling adequacy for factor analysis. Bartlett’s test of sphericity χ^2^ = 3853.615, *p* < 0.001, indicated that items correlated well for factor analysis [43]. Results revealed a three-factor solution that explained 67.35% of the cumulative variance–covariance matrix. The three factors were judged to represent swallowing effort, decline in physical function, and cognitive-communicative difficulties. Results confirmed that all items loaded significantly on to one of those factors. Reliability of each of the three factors was assessed via internal consistency and demonstrated Cronbach’s α > 0.6, indicating good construct validity and reliability (Table 3). 

## 3. Discussion 

Few if any validated pre-clinical screening tools exist to identify potential dysphagia in community dwelling older adults (CDOA). The purpose of this study was to develop and validate a tool to measure early indications of dysphagia in the CDOA incorporating items reported to be related to symptoms of dysphagia in this population. Preliminary data and psychometric analyses support a reliable and valid 17-item tool for the identification of pre-clinical dysphagia in CDOA. The 17-items on the tool consist of questions about swallowing difficulties, similar to those found in other dysphagia tools. However, the addition of questions regarding physical and cognitive–communicative abilities make this tool unique. 

Preliminary factor structure provides some interesting insights into the nature of patient reported pre-clinical symptoms in the CDOA. Data suggests that pre-clinical dysphagia may be primarily influenced by factors related to the functional decline of the older individual—decline in physical abilities and decline in cognitive-communicative abilities. Factors such as weight loss and dental health, traditionally thought to be associated with dysphagia, were not associated with self-report of dysphagia in the CDOA. This observation likely reflects the pre-clinical nature of dysphagia in the study population, as factors such as weight loss may be more relevant in clinical disease with more severe dysphagia. Association of dysphagia with decline in physical abilities and frailty have been reported in several studies. Gonzalez-Fernandez reported significantly increased dysphagia symptoms in CDOA individuals classified as pre-frail based on Fried’s criteria [11]. Likewise, Kawashima reported significant association between abilities to perform ADL’s with dysphagia symptoms [13]. Difficulty in performing ADL’s may be considered a surrogate marker for pre-frail status [44]. In a study on the relationship between swallowing and measures of frailty, Butler and colleagues reported on a specific measure of frailty—i.e., reduced handgrip strength—to be significantly associated with reduced tongue strength. Additionally, they reported that reduced tongue strength was significantly associated with increased aspiration in older adults [8]. A growing body of evidence also suggests that cognitive functioning significantly contributes to functional decline, impact on ADL performance, and subsequent physical decline [24,25,26]. Evidence also suggests that motor function is influenced by cognitive function and cognitive changes affect swallowing, particularly in the oral phase of swallow [45]. In older adults, increased prevalence of physical frailty can increase the risk of cognitive decline and both may combine to have a negative impact on swallowing function. Recent research in dysphagia has also suggested that dysphagia may be part of a geriatric syndrome, associated with sarcopenia and medical and physical decline [46]. These are factors that need to be explored further when considering early identification in CDOA.

Six items across all three factors provided meaningful differentiation between dysphagic and non-dysphagic. These items had moderate-large effect sizes when comparing the two groups. With swallowing, these items included the effort with swallowing solids, pills, and the sensation of food sticking in the throat. Additionally, missing teeth, experience of a dry mouth, and difficulties with everyday activities also differentiated the two groups. This relates to research that has demonstrated associations with these symptoms and swallowing difficulties. While evaluating item performance, non-dysphagic participants perceived most of the items on the survey as more difficult than dysphagic participants. This pattern of response was expected as participants without dysphagia would have more difficulty identifying with questions that ask about swallowing specific issues. This finding therefore underscores the relevance of the questions contained within the survey and provides further insight in to the nature of pre-clinical swallowing symptoms reported by CDOA. 

Early detection of pre-clinical dysphagia may lie in identifying other symptoms that may relate to the development of swallowing difficulties. In the pre-clinical dysphagia screener, this includes symptoms of reduced physical and cognitive function, both of which have been shown to be associated with the development of dysphagia in the CDOA. The addition of these items makes this screening tool different from other dysphagia screeners. Moreover, items that asked about physical and cognitive function are measureable and may provide further insight in to early symptomatology in CDOA. For example, considering the factor of reduced physical function, it was essential to include a question regarding walking ability due to growing consensus that mobility performance is a surrogate marker for overall health and functional ability among older adults [47]. Moreover, the loss of mobility has been predictive of multiple adverse events, including morbidity, worsening of mobility, institutionalization, and mortality [48,49]. Furthermore, standards for measuring items such as walking speed and distance covered have been well established. These accepted measures provide developed physical indices for future research on pre-clinical dysphagia in the CDOA.

A limitation of this study is the participant sample, which included research-interested volunteers suggestive of a volunteer bias. Moreover, these participants were all well-educated. Higher education and access to a computer and Internet is likely indicative of a high socio-economic status (SES). As higher SES is associated with better health status, this suggests yet another source of potential sampling bias [50]. Finally the majority of the sample was women in the 60–70 year age range, impacting generalizability to the CDOA population as a whole, as older CDOA may have increased difficulties and functional decline. 

Future steps in this research will need to include confirmatory factor analysis, comparative analysis with swallowing evaluations, physical ability measures, oral dryness measures, and cognitive measures. Additionally, stability of the test over time and its role in predicting outcomes will need to be assessed. This will not only improve psychometric properties of the screening tool but provide data on the pattern and evolution of swallowing difficulties and related morbidities observed in the CDOA. 

## 4. Implications

The preliminary questionnaire created to evaluate risk of pre-clinical dysphagia in the CDOA reflects the complex symptomology and multi-dimensional nature of swallowing difficulties. The questionnaire demonstrates strong construct validity and high reliability. In a cohort of CDOA individuals, the questionnaire proved to be easy to complete as a PRO, with a 99% completion rate. Furthermore, the questionnaire and analyses provide preliminary insight in to understanding pre-clinical dysphagia in CDOA. Detection of risk for dysphagia may lie in CDOA reporting difficulties with swallowing along with functional decline in performing everyday activities. Collectively, this study indicates that this questionnaire may be a useful screening tool to identify risk for clinical swallowing difficulties in the CDOA. Such a screening tool has a wide range of applications, with the potential for early identification and hence prevention of clinical decline in swallowing-related morbidities of our rapidly aging population.

## Figures and Tables

**Table 1:** Examples of questions in each category. Examples of questions are provided for each of the tool’s categories—swallowing abilities, cognitive abilities, physical abilities, oral health, and social support

Category	Number of Questions	Examples of Questions	Response
Swallowing abilities	11 questions	1. Rate the frequency of the following as they have applied to you in the last month a. I cough when I eat b. When I swallow, food sticks in my throat	Never Rarely Sometimes Often All of the time
		2. Have you lost weight unintentionally in the last 6 months	No weight loss 1–5 pounds 6–10 pounds Greater than 10 pounds
		3. Choose your current diet level (what you eat on a regular basis) from the options presented below	I can eat everything I have to avoid certain foods I can eat soft foods (e.g., scrambled eggs, meatloaf, cooked vegetables) I can only eat puddings/purees (e.g., mashed potatoes, yoghurt, applesauce) in addition to liquids I can only drink liquids
Cognitive abilities	3 questions	1. Rate the frequency of the following as they have applied to you in the last month i. Needed to keep a written list so as to not forget things ii. Had trouble following everyday conversation	Never Rarely Sometimes Often All of the time
Physical abilities	8 questions	1. Rate the frequency of the following as they have applied to you in the last month i. Difficulty dressing yourself ii. Difficulty walking a quarter of a mile ii. Difficulty writing or handling small objects	Never Rarely Sometimes Often All of the time
Oral health	9 questions	1. Do you wear dentures to eat food 2. Are you missing any teeth	Yes No
Social support	3 questions	What is your current living situation?	Live alone Live with spouse/significant other Live with significant other/spouse AND other family members Live with other family members like parents, siblings Live with roommates Live in an assisted living or similar facility
Total number of Questions	34 questions		

**Table 2:** Participant demographics and descriptive data. Participant data results from phase 2 of the study

Variable	% of Participants
Age (60–70 years)	80%
Gender (women)	70%
Education (post-graduate degree)	44%
Living with family	68%
**Swallowing**	
Increased effort with swallowing solids	36%
Increased effort with swallowing pills	21%
Sensation of food getting stuck in throat	42%
Modified diet due to difficulty swallowing	11%
**Other Reported Symptoms**	
Difficulty finding words	43%
Difficulty following everyday conversation	13%
Missing teeth	41%
Experience dry mouth	42%

**Table 3:** Exploratory factor analysis results. Construct validation of the scale completed via exploratory factor analysis (principal components analysis with direct oblimin rotation). Three factors extracted, items in each factor bolded. Factors determined to represent swallowing function, physical function, and cognition.

	Swallowing Effort	Physical Function	Cognitive–Communicative Difficulties
Swallowing solids takes extra effort	0.830		
Swallowing liquids takes extra effort	0.682		
Swallowing pills takes extra effort	0.660		
When I swallow, food sticks in my throat	0.750		
I cough when I eat	0.579		
Swallowing is painful	0.831		
The pleasures of eating is affected by my swallowing	0.939		
Swallowing is stressful	0.932		
I have swallowing problems that interfere with my ability to go out to eat	0.817		
Choose your current diet level (what you eat on a regular basis)	0.749		
Composite of dry mouth questions	0.538		
Difficulty walking a quarter of a mile		0.894	
Difficulty with strenuous activities such as 30 minutes of aerobic activity		0.866	
Difficulty lifting or carrying objects as heavy as 10 pounds		0.869	
QOL interference difficulties with everyday activities		0.857	
Had trouble "finding the right words" when having a conversation			0.840
Had trouble following everyday conversation			0.742
Eigenvalues	7.883	2.428	1.137
Cumulative % of variance	46.371	60.655	67.345
Internal consistency of each factor (Cronbach’s α)	0.901	0.903	0.644
